# Increasing the efficiency of trial-patient matching: automated clinical trial eligibility Pre-screening for pediatric oncology patients

**DOI:** 10.1186/s12911-015-0149-3

**Published:** 2015-04-14

**Authors:** Yizhao Ni, Jordan Wright, John Perentesis, Todd Lingren, Louise Deleger, Megan Kaiser, Isaac Kohane, Imre Solti

**Affiliations:** Cincinnati Children’s Hospital Medical Center, Department of Biomedical Informatics, 3333 Burnet Avenue, MLC 7024 Cincinnati, OH USA; Cancer and Blood Disease Institute, Cincinnati Children’s Hospital Medical Center, Cincinnati, OH USA; Center for Biomedical Informatics, Harvard Medical School, Boston, MA USA; James M Anderson Center for Health Systems Excellence, Cincinnati Children’s Hospital Medical Center, Cincinnati, OH USA

**Keywords:** Automated clinical trial eligibility screening, Patient-trial matching, Natural language processing, Information extraction

## Abstract

**Background:**

Manual eligibility screening (ES) for a clinical trial typically requires a labor-intensive review of patient records that utilizes many resources. Leveraging state-of-the-art natural language processing (NLP) and information extraction (IE) technologies, we sought to improve the efficiency of physician decision-making in clinical trial enrollment. In order to markedly reduce the pool of potential candidates for staff screening, we developed an automated ES algorithm to identify patients who meet core eligibility characteristics of an oncology clinical trial.

**Methods:**

We collected narrative eligibility criteria from ClinicalTrials.gov for 55 clinical trials actively enrolling oncology patients in our institution between 12/01/2009 and 10/31/2011. In parallel, our ES algorithm extracted clinical and demographic information from the Electronic Health Record (EHR) data fields to represent profiles of all 215 oncology patients admitted to cancer treatment during the same period. The automated ES algorithm then matched the trial criteria with the patient profiles to identify potential trial-patient matches. Matching performance was validated on a reference set of 169 historical trial-patient enrollment decisions, and workload, precision, recall, negative predictive value (NPV) and specificity were calculated.

**Results:**

Without automation, an oncologist would need to review 163 patients per trial on average to replicate the historical patient enrollment for each trial. This workload is reduced by 85% to 24 patients when using automated ES (precision/recall/NPV/specificity: 12.6%/100.0%/100.0%/89.9%). Without automation, an oncologist would need to review 42 trials per patient on average to replicate the patient-trial matches that occur in the retrospective data set. With automated ES this workload is reduced by 90% to four trials (precision/recall/NPV/specificity: 35.7%/100.0%/100.0%/95.5%).

**Conclusion:**

By leveraging NLP and IE technologies, automated ES could dramatically increase the trial screening efficiency of oncologists and enable participation of small practices, which are often left out from trial enrollment. The algorithm has the potential to significantly reduce the effort to execute clinical research at a point in time when new initiatives of the cancer care community intend to greatly expand both the access to trials and the number of available trials.

**Electronic supplementary material:**

The online version of this article (doi:10.1186/s12911-015-0149-3) contains supplementary material, which is available to authorized users.

## Background

Although several reports have described positive experiences leveraging electronic health record (EHR) information to facilitate trial recruitment, eligibility screening (ES) is still conducted manually in most cases [1-3]. Manual ES typically requires a lengthy review of patient records and trial criteria descriptions, a cumbersome process that creates a significant financial burden for an institution [4]. The clinical trial phase is the most expensive component of drug development; therefore, any improvement in the efficiency of the recruitment process should be highly consequential [5]. The factor that most clinical practices are not staffed for manual patient screening is also a challenge for clinical trial recruitment. For these reasons, automatically prescreening and identifying trial-patient matches, on the basis of EHR information, promises great benefits for translational research.

Several informatics tools have been described in the literature to automate trial-patient matching [6-19]. There are two approaches to matching patients and trials: 1) identifying a cohort of patients for a particular trial; and 2) identifying clinical trials for an individual patient. In this study, we refer to the first use case as *trial-centered patient cohort identification* and the second as *patient-centered trial recommendation*. Recent studies mainly focus on the first approach because they usually target a small set of clinical trials [6,7,10-12,15-17]. Nevertheless, patient-centered trial recommendation is also valuable, particularly if the key barrier to physician participation is the time required for identifying appropriate trials for individual patients from a large pool of active trials [18].

Despite these previous efforts, many barriers remain for automated ES [20,21]. Early studies were dedicated to matching trial eligibility criteria with structured and semi-structured EHR data (e.g. demographics, ICD-9 codes and laboratory results) [6,7,9,15]. However, the logic-based triggers usually require manual design. Automatically generating computable triggers from narrative eligibility criteria remains challenging [22]. On the other hand, since a substantial portion of meaningful information in the EHR is represented only in narrative text, progress in natural language processing (NLP) and information extraction (IE), can enhance the accuracy of trial-patient matching [21,23]. Relevant NLP and IE techniques have been summarized and reported in the annual Text Retrieval Conference (TREC) medical record track [21,24-30]. However, only a handful of the techniques were evaluated on real-world trial-patient matching, and most of them focused on one clinical trial [10-12,14,16]. Even the TREC medical record track, because of the lack of available real-world trial-patient matches, had to use synthetic clinical queries to evaluate ES algorithms. Additional study of the algorithms is therefore required to address the gap in evaluation.

We implemented and developed an automated ES algorithm in our earlier pediatric Emergency Department (ED) study [17]. The algorithm consisted of three core components: 1) a logic-based filter that excluded patients based on EHR structured data fields, 2) an NLP-based concept detector that identified keywords and medical terms from narrative eligibility criteria and patient clinical notes, and 3) an IE-based trial-patient matching function that computed the degree of match between trial criteria and patient clinical information (see “Automated ES algorithm” for detailed implementation). The algorithm has been evaluated retrospectively on all clinical trials that recruited patients with specific diagnoses in our ED during the study period, and it showed promising performance in trial-centered patient cohort identification [17]. The objective of the current study is to validate the generalizability of the ES algorithm on real-word pediatric oncology clinical trials and EHR data, where the specific aim is to identify patients who meet core eligibility characteristics for cancer trials. Due to the large number of trials available to a pediatric oncology patient, patient-centered trial recommendation becomes important in oncology trial recruitment [18]. Therefore, in this study we evaluate the proposed algorithm on both trial-centered and patient-centered scenarios.

## Methods

The study population consisted of all pediatric oncology patients admitted at Cincinnati Children’s Hospital Medical Center (CCHMC) between December 2009 and October 2011. Approval of ethics for this study was given by the CCHMC institutional review board (study ID: 2010–3031) and a waiver of consent was authorized.

### Clinical trials and data

#### Eligibility criteria descriptions of clinical trials

We composed a comprehensive list of the 70 oncology trials, which enrolled patients at CCHMC during the study period. To be more conservative in the evaluation of the ES algorithm, we excluded all repository trials, which customarily enrolled all patients, and the institutional trials for which we did not find the trial announcements on ClinicalTrials.gov. This process resulted in a set of 55 trials for the current study.

**Figure 1:**
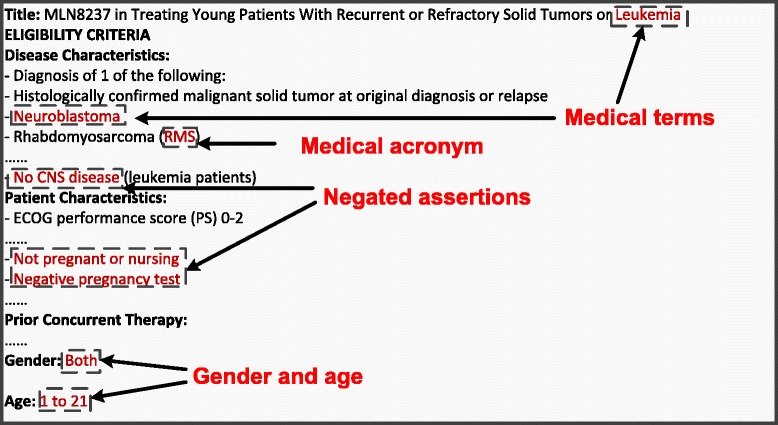
An example eligibility description (NCT01154816) derived from ClinicalTrials.gov.

To obtain the narrative eligibility criteria of the trials, we searched their NCT identifiers on ClinicalTrials.gov and downloaded the corresponding inclusion and exclusion sections. The list of the trials including NCT identifiers, number of enrolled patients during the study period, opening and closing dates, and special circumstances in enrollment are presented in (Additional file 1: Table S1). Figure 1 shows an example eligibility criteria section. Compared with the clinical trials used in our earlier study [17], the criteria of oncology trials were more descriptive and contained more disease-related terms and acronyms (Figure 1). In addition, two demographic attributes, age and gender, were retrieved from the eligibility criteria via NLP techniques.

#### Patient EHR data

**Figure 2:**
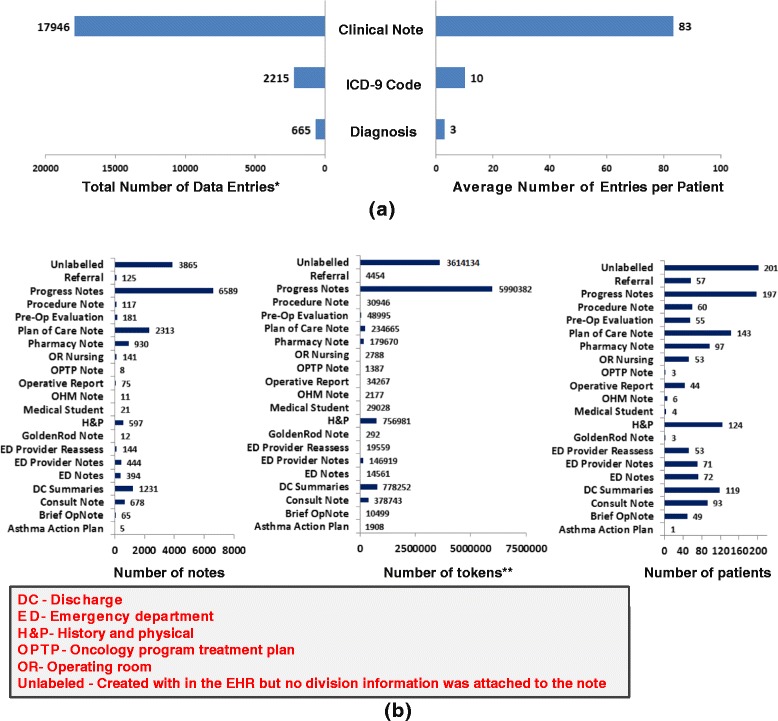
Frequencies of the collected EHR fields **(a)** and descriptive statistics of the unstructured clinical notes **(b)**. *A data entry is a piece of information (e.g. diagnosis) documented during a patient’s visit. If a patient has the same diagnosis/ICD-9 code during multiple visits, we only count the diagnosis/ICD-9 code once for that patient. **Tokens include words, numbers, symbols and punctuations in clinical narratives.

During the study period 215 CCHMC patients participated in cancer treatment and all of them were included in our study. Based on the trials’ criteria, we extracted the demographics (age and gender), diagnoses and associated ICD-9 codes from structured EHR data, and unstructured clinical notes to represent the patients’ profiles. Figure 2 shows the frequencies of the collected EHR data fields and the descriptive statistics of the clinical notes. The structured diagnoses and ICD-9 codes contained precise information about the patients’ clinical problems, while the unstructured clinical notes provided more comprehensive information including symptoms and disease progression. Compared with the ED patients investigated in our earlier study [17], the pediatric oncology patients had more diagnoses and clinical notes.

Because some of the diagnoses and notes were entered in the EHR after the end of a trial’s enrollment period, when automating the ES for that trial, we excluded them if they had an EHR entry-timestamp after the trial's closing date. Furthermore, if his/her physician enrolled a patient in a trial, we only used the diagnoses and notes written before the patient’s enrollment date in that particular trial. The information collected until that point represented the information that was available to the physician at the time of making the enrollment decision.

#### Historical trial-patient enrollment decisions

One hundred and twenty seven patients were enrolled in one or more of the 55 trials, providing us with 169 patient-trial matches as a reference standard. Unlike for adult clinical trials, the enrollment of pediatric oncology patients is almost universal. Almost all eligible patients accept trial invitations. The National Cancer Institute (NCI) bulletin shows that more than 90% of pediatric oncology patients younger than five participate in trials [31]. The enrollment rate is lower in adolescents but it is still a magnitude higher than in adults.

The special circumstances of pediatric oncology trial screening have two important consequences. First, although the historical enrollment decisions do not build a traditional gold standard because they were not made as part of a controlled double chart review process (e.g., inter-screener agreement is not available); they produce a useful reference standard to evaluate the ES algorithm because the decision making covers the entire study population. Second, because of the high enrollment rate specific for the study population, to determine the generalizability of conclusions drawn from testing the algorithm on retrospective pediatric oncology trial enrollment decisions will require additional research.

### Automated ES algorithm

**Figure 3:**
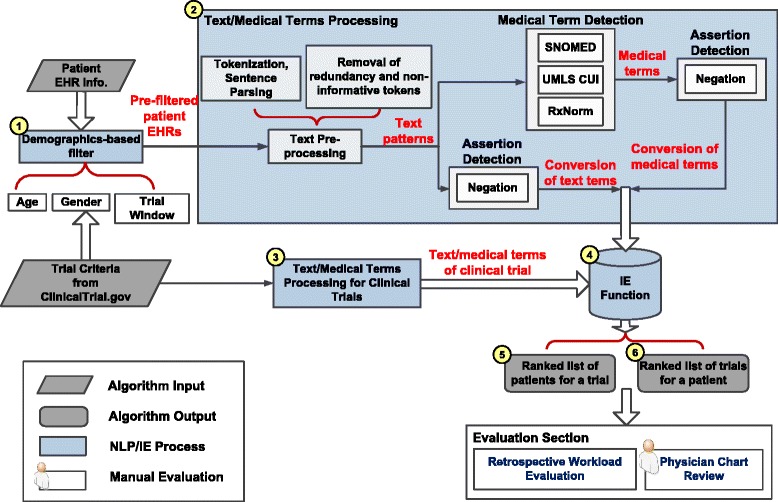
The architecture of the automated ES algorithm.

We customized and implemented state-of-the-art NLP and IE techniques to build the automated ES architecture [17,24-30]. In trial-centered patient cohort identification, a logic-based filter was applied first utilizing demographics to exclude ineligible patients for a trial (Step 1 in Figure 3). The diagnoses and clinical notes of pre-filtered patients were then processed, from which the medical terms were extracted and stored in the patient pattern vectors (Step 2). The same process was applied to the trial criteria to construct the trial pattern vector (Step 3). Finally, the IE function computed the degree of match between the patient vectors and the trial pattern vector (Step 4) and output the ranked list of patients based on the matching scores (Step 5). Vice versa, the ES algorithm also output a ranked list of trials for a patient in patient-centered trial recommendation (Step 6).

#### Demographics-based filter

Since age and gender were retrieved from the eligibility criteria (Figure 1), we adopted them as demographics-based filters that have been proven to be beneficial by earlier studies [24-26]. We also applied the trial enrollment window to facilitate the pre-filtering process. If a patient did not have clinical notes within the enrollment window of a trial (e.g. between the opening and the closing dates), implying that patient was not participating in the care of CCHMC physicians during the enrollment period, the patient was ruled out for that particular trial.

#### Text and medical terms processing

The text and medical terms processing utilized advanced NLP techniques to extract medically relevant information from the patients’ clinical notes and the trial eligibility descriptions. Details of the NLP process can be found in our earlier publications [17,32-35]. To summarize, the algorithm first extracted text-driven, term-level medical information (e.g. keywords and acronyms in Figure 1) from clinical narratives using the Apache clinical Text Analysis and Knowledge Extraction System (cTAKES) [36]. cTAKES then assigned medical terms to the identified text strings from controlled terminologies, including Concept Unique Identifiers (CUI) from the Universal Medical Language System (UMLS), the Systematized Nomenclature of Medicine Clinical Terms codes (SNOMED-CT), and a standardized nomenclature for clinical drugs (RxNorm) [37-39]. The same process was applied to identify text and medical terms from the diagnosis strings. In addition, the ICD-9 codes were mapped to SNOMED-CT terms using the UMLS ICD-9 to SNOMED-CT dictionary [40].

To identify negations, we implemented a negation detector based on the NegEx algorithm [41]. For example, the phrase “No CSN disease” (Figure 1) was converted to “NEG23853001”. The text and medical terms were converted if necessary in the assertion detection component. Finally, all identified text and medical terms were stored as bag-of-words in a patient vector.

For the trial eligibility description, the same text and medical term processing was applied to the inclusion and exclusion criteria to extract term-level patterns. All terms extracted from the exclusion criteria were converted into negated format.

#### Trial-patient matching function

The text and medical terms extracted from a patient’s EHR were stored in a vector to represent the patient’s profile. The same process was executed to build the pattern vector for a clinical trial. The IE function then matched the trial and the patient vectors and computed the matching score for each trial-patient pair [42]. Finally, a ranked list of patient candidates was returned for a trial in trial-centered patient cohort identification and a ranked list of trials for a patient in patient-centered trial recommendation.

### Experiments

We used two methods to evaluate the performance of the ES algorithm. First, we evaluated the screening efficiency in identifying all historical enrollment decisions shown in Table 1A. We refer to this evaluation as retrospective workload evaluation. Second, an oncologist performed a manual review of the algorithm's randomly selected 76 trial-patient assignments. We refer to this evaluation as physician chart review.

**Table 1:** **The performance of the demographics-based filter (baseline) and the EHR-based ES algorithms**

**Trial-centered Patient Cohort Identification**										
**Algorithm**	**Evaluation Metrics**									
	**WL**	**95% CI**	**P[%]**	**Sp[%]**	PV*					
Demographics-based Filter	163	149-179	1.9	24.3	8.30E-21					
DX/ICD-9	50	35-64	6.20	78.1	5.27E-4					
NOTE	28	16-41	10.7	87.9	7.75E-2					
DX/ICD-9+NOTE	24	14-35	12.6	89.9	N/A					
**Patient-centered Trial Recommendation**										
**Algorithm**	**Evaluation Metrics**									
	**Sub-population case (127 patients)**					**Full-population case (215 patients)**				
	**WL**	**95% CI**	**P[%]**	**Sp[%]**	PV*	**WL**	**95% CI**	**P[%]**	**Sp[%]**	PV*
Demographics-based Filter	42	40-43	3.2	25.5	1.7E-143	42	40-43	1.9	24.3	1.5E-39
DX/ICD-9	8	6-10	16.8	87.8	2.36E-7	22	19-25	3.6	60.7	3.85E-7
NOTE	4	3-5	33.1	95.0	2.54E-2	20	17-23	3.9	64.9	2.54E-2
DX/ICD-9+NOTE	3	3-4	35.7	95.5	N/A	19	17-22	4.0	65.5	N/A

DX/ICD9 indicates ES algorithm using only structured diagnoses and ICD-9 codes; NOTE, ES algorithm using only clinical notes; DX/ICD-9+NOTE, ES algorithm using both structured data and clinical notes.

WL indicates workload; CI, confidence interval; P, precision and Sp, specificity, PV, p-value.

*P-values were calculated by comparing the workload between DX/ICD-9+NOTE with the other algorithms.

N/A indicates that the performances between the two algorithms are identical and no p-value is returned.

For comparison, we used the output of the demographics-based filter, which has been implemented in many EHR products, as the baseline. In trial-centered patient cohort identification, the baseline excluded ineligible patients by demographics and randomly shuffled the rest of the candidates for a trial. Similarly, it excluded ineligible trials and randomly shuffled the pre-filtered trials for a patient in patient-centered trial recommendation. The baseline simulated the screening process without automated ES, replicating current practice. In addition, we validated the contributions of the structured diagnoses/ICD-9 codes and the unstructured clinical notes. That is, we used the two data sets individually and in combination in the ES algorithm and assessed the performance respectively.

#### Retrospective workload evaluation

To assess the screening efficiency of the algorithms, we calculated the average workload of the recruitment process [16]. In trial-centered patient cohort identification, the workload is defined as the number of patients an oncologist would be required to review, from the population of 215 patients, to identify all patients historically enrolled in a particular trial.

In patient-centered trial recommendation, the number of trials an oncologist would need to review to replicate a patient’s historical trial enrollments defines the workload. For this scenario, the algorithm was evaluated on the 127 patients who had historical enrollments. We refer to this result as sub-population case. In practice some patients (e.g. patients who did not have historical enrollments) could be ineligible for all clinical trials and an oncologist would have to screen all available trials to confirm their ineligibity. To assure the integrity of the evaluation, we also evaluated the algorithms on all 215 patients, which we refer to as the full-population case. Note that in this case, an oncologist would have to review all algorithm output trials to confirm a patient’s ineligibility if the patient had no historical enrollments (88 patients in the study).

In addition to the average workload, precision (denoted by P) and specificity (denoted by Sp) were applied to measure screening performance. Since the goal of the retrospective workload evaluation was to identify all historical enrollments (i.e. False Negative = 0), the recall = True Positive/(True Positive + False Negative) and the negative predictive value NPV = True Negative/(True Negative + False Negative) were always 100%.

#### Physician chart review

An oncologist conducted a manual, retrospective, chart review on a randomly selected set of charts to determine the ES algorithm’s precision more realistically, because the historical enrollment decisions might have depended on factors that were not included in the scope of this study. For example, we did not try to detect the patients’ preferences for a particular cancer treatment, for outpatient versus inpatient care, or for the route of administration (e.g. pill versus infusion therapy). Our ES algorithm might predict candidate trials for a patient that the patient was truly eligible for, based on his/her clinical information, but because of the patient’s preference the physician did not enroll the patient in that trial. Consequently, the retrospective workload evaluation would report a lower algorithm precision than the true measure would be based on the scope of our study. Adding the manual retrospective chart review accounted for the external factors (e.g. patient preferences described in the clinical notes) and provided an adjustment for the precision.

We reported the results of the physician chart review on trial-centered patient cohort identification. Specifically, we randomly sampled ten trials (20% of the trials) and regarded the top 2 × N candidates from the ES algorithm as potentially eligible patients, where “N” denotes the number of actually enrolled patients for a particular trial at CCHMC (Table 1A). For instance, the NCT00134030 trial had nine historical enrollments and the ES algorithm provided 18 patients as potentially most eligible candidates for the trial. In the next step an oncologist with clinical trial experience reviewed the clinical notes, which were written during the study period, and determined how many of the 18 patients were truly eligible for the NCT00134030 trial. Finally, the precision of the ES algorithm was re-calculated based on the results of the chart review. The physician chart review also contributed information to our error analysis and identified limitations of the automated ES algorithm.

**Table 2:** **The precision of the ES algorithm against the historical enrollments and the list of eligible patients found by the oncologist**

**NCT ID**	**Number of historical enrollments for the trial (N)**	**Number of algorithm outputs (2 × N)**	**Number of historical enrollments in the algorithm outputs**	**Number of additional eligible patients found by the oncologist in the algorithm outputs**
NCT00072384	1	2	1	0
NCT00134030	9	18	9	2*
NCT00274937	1	2	1	0
NCT00335556	2	4	0	0
NCT00343694	3	6	1	1*
NCT00379340	1	2	0	0
NCT00382109	2	4	0	0
NCT00553202	6	12	4	1*
NCT00557193	1	2	1	0
NCT01190930	12	24	12	1*
TOTAL	38	76	29	5
Precision	N/A	N/A	0.38	0.45

*Indicates that more patients in the algorithm output were eligible for this trial than the number of historical enrollment decisions.

## Results

### Retrospective workload evaluation

Table 1 shows the results of eligibility screening with the baseline (demographics-based filter) and the EHR-based ES algorithms. For trial-centered patient cohort identification, an oncologist would need to review 163 patients per trial using the baseline. Utilizing structured diagnoses/ICD-9 codes in the ES algorithm (DX/ICD-9), the workload was reduced to 50 patients per trial. By leveraging both structured data and clinical notes, the ES algorithm (DX/ICD-9 + NOTE) achieved the best workload performance (24 patients per trial), amounting to an 85% workload reduction over the baseline (p-value = 8.30E-21 in paired-T test).

In automated patient-centered trial recommendation, we observed consistent improvement in workload when more EHR data was used. Compared with the baseline, the workload was reduced by more than 90% to 4 trials per patient when the complete ES algorithm (DX/ICD-9 + NOTE) was leveraged. Even for the full-population case, the average workload of automated ES was statistically significantly lower than the baseline (20 versus 42 trials per patient, p-value = 1.5E-39 in paired-T test).

### Physician chart review

Table 2 shows the findings of the physician chart review. From the list of algorithm generated patient candidates the oncologist found that 34 patients were truly eligible for the ten randomly selected trials. On the other hand, the retrospective data showed only 29 enrolled patients. Consequently, the adjusted precision of the ES algorithm increased to 0.45 on this randomly sampled trial-patient set, amounting to an 18.4% relative improvement over the 0.38 precision against the historical enrollment data.

**Table 3:** **The false positive errors made by the ES algorithm with the causes described by the oncologist**

**Cause of false positive errors identified by the physician chart review**	**Number of errors**
Previously enrolled in the trial/therapy at a different institution	3
New diagnosis treated with standard of care therapy due to high likelihood of survival	4
Correct diagnosis but in a different stage of the disease (e.g. high risk versus low risk)	5
Correct diagnosis but incorrect relapse status (e.g. relapsed versus non-relapsed, remission 1 versus remission 2)	5
Wrong diagnosis, confusion between sub-categories of diseases (e.g. ALL versus AML, T cell versus Pre-B cell and different types of renal tumors)	13
Wrong diagnosis, other reasons	12

## Discussion

### Performance analysis

Our results show that a fully-automated ES algorithm, that relies on the narrative eligibility criteria of clinical trials and the information from patient EHRs, could achieve notable workload reduction in both trial-centered patient cohort identification (85%) and patient-centered trial recommendation (more than 90%) compared with demographics-based screening (Table 1). The ES algorithm showed good capability in matching the descriptive criteria with patients’ clinical problems (DX/ICD-9 results in Table 1). However, without the comprehensive information from clinical notes (Figure 2), the algorithm would be unable to achieve accurate trial-patient matching. Using clinical notes (NOTE) greatly improved the matching accuracy of the ES algorithm, which was evidenced by a workload reduction of 44% over the DX/ICD-9 algorithm in trial-centered patient cohort identification and 50% in patient-centered trial recommendation. In addition, the NOTE algorithm’s performance was close to the performance of the ES using both structured data and clinical notes (DX/ICD-9 + NOTE). This is expected because the patients’ diagnoses were also documented in the clinical notes (e.g. History & Physical notes and discharge summaries). Consequently, the structured diagnosis/ICD-9 data did not contribute substantially when clinical notes were used. The observations validate the value of unstructured clinical notes in automated ES and confirm the effectiveness of the NLP and IE techniques as previously demonstrated by us and other groups [17,24,25,29].

Projecting the results of the physician chart review to the entire data set, the performance of the automated ES algorithm would be improved by 18.4% to 0.149 (precision) in trial-centered patient cohort identification. Further refinements of the algorithm are required to increase precision. However, even at this early stage of development, automated ES provides a sufficiently high yield of accurate screening hits to substantially improve the oncologists’ efficiency in evaluating the patients’ eligibility for clinical trials.

### Error analysis, limitations and future work

To describe the limitations of our ES algorithm, we grouped the causes of the 42 oncologist-identified errors into six categories (Table 3). The error analysis suggested several areas for improvement. First, 54.7% of the errors (categories 3–5) were caused because the algorithm confused similar phrases (e.g. “T cell lymphoblastic lymphoma” versus “Pre-B cell lymphoblastic lymphoma”). The reason is that our algorithm used individual words as patterns, limiting its ability in finding semantic relations between consecutive words. In the future we will integrate advanced NLP techniques to analyze semantic relations to see if they improve the accuracy of medical concept identification. Second, the algorithm failed to distinguish the patients’ new and historical diagnoses and caused an additional 10% of the errors (category 2). The observation validated the need for including temporal reasoning in automated ES. Finally, the logic-based filter was restricted to screening the demographics only, which limited its ability in capturing certain exclusion criteria (e.g., previous enrollment status, category 1). The algorithm will be more powerful if more information from the structured data fields (e.g. enrollment information, vital signs and laboratory results) of the EHR are included. The steps to extract this information from narrative eligibility criteria to design a complex logic-based filter will also be investigated in future works.

One limitation of our study is that the evaluation was restricted to retrospective data. Project planning is in progress to evaluate the practicality of the automated ES in a randomized controlled prospective test environment. In addition, the study’s scope was restricted to pediatric oncology clinical trials. Because of the almost universal clinical trial enrollment in pediatric oncology, it is possible that the patients’ notes include more information to automate the ES than the adult oncology patients’ notes would. To verify the transferability of the findings, we also plan to test the ES algorithm on a more diversified oncology patient population (e.g. adult patients) and include multiple institutions. Finally, the text of the eligibility criteria in oncology trials is more descriptive than in other subspecialties, providing a potentially more suitable foundation for NLP and IE. If a trial's eligibility criteria involve more quantitative thresholds, a different type or a hybrid approach involving quantitative logic and NLP/IE may be more appropriate, as we showed in an earlier study [17].

## Conclusion

By leveraging NLP and IE technologies on both the trial criteria and the EHR content of the patients, an automated eligibility pre-screening algorithm could dramatically increase the trial screening efficiency of oncologists. In a retrospective evaluation of real world data, the algorithm achieved 85% workload reduction in trial-centered patient cohort identification and more than 90% in patient-centered trial recommendation, while keeping the precision at a manageable level (12.6% and 35.7% respectively). Consequently, we hypothesize that the algorithm, when rolled out for production, will have the potential to substantially reduce the time and effort necessary to execute clinical research, particularly as important new initiatives of the cancer care community (e.g. the NCI National Clinical Trials Network) intend to greatly expand both the access to trials and the number of available trials.

The study also demonstrated the usability of the physicians’ historical enrollment decisions for evaluating automated ES algorithms. However, the results showed the need for manual chart review to determine the true level of algorithm precision when such evaluation is conducted.

## Additional file

Additional file 1: Table S1. The list of clinical trials and the corresponding numbers of eligible patients in the reference standard set.

## Abbreviations

CCHMC: Cincinnati Children’s Hospital Medical Center
CI: Confidence Interval
cTAKES: clinical Text Analysis and Knowledge Extraction System
CUI: Concept Unique Identifier
EHR: Electronic Health Record
ES: Eligibility Screening
IE: Information Extraction
NCI: National Cancer Institute
NLP: Natural Language Processing
NPV: Negative Predictive Value
P: Precision
PV: p-value
SNOMED-CT: Systematized Nomenclature of Medicine Clinical Terms codes
Sp: Specificity
UMLS: Universal Medical Language System
WL: Workload
