# Supplementary material for: Increasing the efficiency of trial-patient matching: automated clinical trial eligibility Pre-screening for pediatric oncology patients
Source: BMC Med Inform Decis Mak. 2015 Apr 14;15:28. doi: 10.1186/s12911-015-0149-3 (PMC4407835; doi:10.1186/s12911-015-0149-3)
Supplement: Additional file 1: Table S1. — The list of clinical trials and the corresponding numbers of eligible patients in the reference standard set. [file 12911_2015_149_MOESM1_ESM.docx]

**Additional file 1**

**Table S1** The list of clinical trials and the corresponding numbers of eligible patients in the reference standard set.

| **NCT ID** | **#Enrollments** | **Phase** | **Opening date** | **Closing Date** | **NOTE** |
| --- | --- | --- | --- | --- | --- |
| NCT00072384 | 1 | 3 | 1/22/2008 | 1/28/2011 |  |
| NCT00091091 | 1 | No Phase Specified | 9/8/2006 | 6/27/2011 |  |
| NCT00103285 | 2 | 3 | 5/17/2005 | 5/28/2010 |  |
| NCT00134030 | 9 | 3 | 6/29/2006 | 6/27/2011 |  |
| NCT00274937 | 1 | 3 | 8/21/2006 | active |  |
| NCT00295919 | 2 | 1 | 8/9/2005 | active |  |
| NCT00304070 | 1 | 3 | 2/7/2007 | active |  |
| NCT00304083 | 2 | 2 | 7/1/2008 | 7/1/2012 |  |
| NCT00335556 | 2 | 2 | 8/24/2006 | active |  |
| NCT00343694 | 3 | 1 | 8/2/2006 | 3/16/2012 |  |
| NCT00346164 | 5 | 3 | 8/17/2007 | 2/6/2012 |  |
| NCT00352534 | 2 | 3 | 3/2/2007 | active |  |
| NCT00372593 | 1 | 3 | 10/17/2006 | 6/15/2010 |  |
| NCT00379340 | 1 | 3 | 8/17/2007 | active |  |
| NCT00381680 | 1 | 3 | 9/5/2007 | active |  |
| NCT00382109 | 2 | 3 | 9/25/2007 | 5/11/2011 |  |
| NCT00387920 | 1 | 1 | 10/25/2006 | 3/18/2011 |  |
| NCT00408005 | 3 | 3 | 6/15/2007 | active |  |
| NCT00482352 | 7 | No Phase Specified | 1/5/2005 | 9/6/2011 |  |
| NCT00553202 | 6 | 2 | 2/5/2008 | active |  |
| NCT00557193 | 1 | 3 | 4/21/2008 | active |  |
| NCT00567567 | 1 | 3 | 12/14/2007 | 2/27/2012 |  |
| NCT00572182 | 3 | 1 | 11/12/2008 | 2/18/2011 |  |
| NCT00590915 | 2 | No Phase Specified | 5/22/2007 | 11/18/2011 |  |
| NCT00666588 | 1 | 2 | 6/5/2008 | 1/17/2012 |  |
| NCT00671034 | 1 | No Phase Specified | 11/6/2008 | 9/4/2012 |  |
| NCT00720109 | 1 | 3 | 9/10/2008 | 2/6/2012 |  |
| NCT00742924 | 1 | No Phase Specified | 11/24/2008 | 2/9/2012 |  |
| NCT00786669 | 5 | 1, 2 (multiple phases) | 2/29/2008 | 11/2/2011 | Suspended from 9/1/2010 to 10/14/2010 |
| NCT00866918 | 1 | 3 | 4/30/2009 | 11/12/2012 | Suspended from 9/24/2012 to 10/10/2012 |
| NCT00880282 | 4 | 1 | 5/7/2009 | 3/9/2012 |  |
| NCT00883688 | 1 | 2 | 9/3/2009 | 2/1/2013 |  |
| NCT00898365 | 12 | No Phase Specified | 6/13/2006 | active |  |
| NCT00904241 | 8 | No Phase Specified | 3/6/2001 | active |  |
| NCT00919269 | 14 | No Phase Specified | 7/1/1999 | active |  |
| NCT00929903 | 3 | 1 | 8/3/2009 | 8/19/2011 |  |
| NCT00933985 | 1 | 1 | 7/24/2009 | 11/9/2012 |  |
| NCT00939770 | 2 | 1, 2 (multiple phases) | 10/17/2009 | active |  |
| NCT00945009 | 1 | 3 | 10/7/2009 | active |  |
| NCT00980460 | 5 | 3 | 10/7/2009 | active |  |
| NCT00994500 | 2 | 1 | 11/9/2009 | 3/11/2011 |  |
| NCT01019850 | 2 | 1 | 3/15/2010 | 3/3/2012 | Suspended from 5/16/2011 to 9/3/2011 |
| NCT01026220 | 1 | 3 | 6/8/2010 | 1/19/2012 |  |
| NCT01041638 | 3 | 3 | 2/22/2010 | 5/11/2011 |  |
| NCT01055314 | 1 | No Phase Specified | 2/19/2010 | active |  |
| NCT01076530 | 3 | 1 | 4/26/2010 | 1/14/2011 |  |
| NCT01141244 | 1 | 1 | 8/6/2010 | active |  |
| NCT01142427 | 11 | No Phase Specified | 9/14/2010 | active |  |
| NCT01154816 | 2 | 2 | 4/25/2011 | active |  |
| NCT01164163 | 1 | 1 | 9/22/2010 | active |  |
| NCT01169584 | 4 | 1 | 10/28/2010 | 12/29/2012 |  |
| NCT01190930 | 12 | 3 | 9/16/2010 | active |  |
| NCT01231906 | 1 | 3 | 2/2/2011 | active |  |
| NCT01231919 | 2 | 1 | 1/5/2011 | 7/27/2012 |  |
| NCT01240538 | 2 | 1 | 3/30/2011 | active |  |

“active” indicates that a clinical trial is actively enrolling patients.
